# Reflective practice to reduce moral injury in healthcare workers

**DOI:** 10.1093/occmed/kqag060

**Published:** 2026-09-17

**Authors:** S Tazrin, T Niyarepola, S A M Stevelink, R Bhundia, D Lamb, N Greenberg, B Croak

**Affiliations:** Department of Psychology, King’s College London, London, SE5 8AF, UK; Department of Psychology, King’s College London, London, SE5 8AF, UK; King’s Centre for Military Health Research, King’s College London, London, SE5 8AF, UK; Department of Psychological Medicine, King’s College London, London, SE5 8AF, UK; King’s Centre for Military Health Research, King’s College London, London, SE5 8AF, UK; Department of Psychological Medicine, King’s College London, London, SE5 8AF, UK; Primary Care and Population Health, UCL, London, WC1E 7HB, UK; King’s Centre for Military Health Research, King’s College London, London, SE5 8AF, UK; Department of Psychological Medicine, King’s College London, London, SE5 8AF, UK; King’s Centre for Military Health Research, King’s College London, London, SE5 8AF, UK; Department of Psychological Medicine, King’s College London, London, SE5 8AF, UK

## Abstract

**Background:**

Moral injury (MI) can arise when individuals experience events that violate their moral beliefs. During the COVID-19 pandemic, MI gained attention among healthcare workers (HCWs). Reflective practice (RP) may help prevent or mitigate MI but remains underexplored. This narrative review examines existing literature on RP interventions addressing MI in HCWs.

**Aims:**

To synthesize existing literature on RP interventions to prevent or mitigate MI in HCWs.

**Methods:**

A semi-structured literature search was conducted in July 2024 and updated in August 2025 across four databases. Twelve studies evaluating RP interventions, including three studies identifying barriers to RP, were included. Across the 12 studies, the sample size was *n* = 793, involving nurses and other HCWs.

**Results:**

Most studies implemented structured workshops combining RP with psychoeducation and group discussion. Of the studies, 11 studies lacked control groups, three used pre-/post-test designs, while others relied on self-reported helpfulness. These studies provided preliminary evidence for the feasibility and perceived value of RP in addressing MI. The only study with a control group was limited by a small sample size, and the results were non-significant. Reported barriers to implementing RP included individual-level factors (e.g. lack of experience) and institutional obstacles (e.g. limited managerial support).

**Conclusions:**

Findings suggest RP holds promise as an intervention for MI in HCWs. However, the evidence base is limited by small samples and weak study designs. Further research using robust methodologies, including randomized controlled trials (RCTs) and longitudinal studies, is needed to evaluate RP across diverse HCW roles, including administrative staff.

Key learning points **What is already known about this subject:**

- Moral injury, a mental health syndrome which can follow after a violation of one’s moral code, has primarily been studied in military personnel.
- The aftermath of the COVID-19 pandemic exacerbated existing systemic issues for healthcare workers, including understaffing, lack of resources and inadequate Personal Protective Equipment; thus, healthcare workers may have been exposed to potentially morally injurious events and experienced moral injury as a result.
- Reflective practice may be a feasible, yet under-researched method of mitigating moral injury in healthcare workers.

**What this study adds:**

- We found 12 studies that investigated reflective practice as an intervention for moral injury in healthcare workers. Eight of the studies were with nurses, and the remaining four were with a diverse range of healthcare workers.
- Eleven studies did not include a control group but suggested preliminary support for reflective practice as a useful intervention for healthcare workers through self-reports based on a pre/post-test experimental design; the one study using a control group found no statistically significant differences in moral injury scores following the intervention.
- Three studies investigated barriers to engaging in reflective practice for healthcare workers and found that barriers can be grouped as being personal (such as lack of familiarity with reflective practice) and institutional (such as lack of managerial support).

**What impact this may have on practice or policy:**

- Although there is preliminary support for reflective practice as an intervention for moral injury in healthcare workers, the evidence base is in its infancy; further research with more methodologically robust techniques, including longitudinal approaches and randomized controlled trials, is necessary to establish the evidence base for reflective practice as an intervention.
- The research on barriers suggests that systemic change is necessary to enable healthcare workers to engage in reflective practice; even identified personal barriers (such as lack of familiarity and time) were a result of the systemic pressures in healthcare settings.
- Integrating better preparation for potentially morally injurious events in medical training and training for all staff (including clinical and non-clinical) should be implemented in healthcare.

## Introduction

Moral injury (MI) is an emotional, behavioural, cognitive and physical response which follows exposure to events that violate someone’s core moral or ethical code [1]. Such events are often referred to as potentially morally injurious events (PMIEs), which include acts of commission (e.g. actions contrary to a person’s belief system), omission (e.g. failures to act in line with one’s own belief system) and feeling betrayed, often by a trusted other [2]. Currently, MI is not a formal psychiatric diagnosis but rather a syndrome [3], associated with intense reactions such as shame, guilt, contempt or anger [1]. A related term is moral distress (MD), defined as discomfort following PMIEs. While MD and MI involve ethical violations, MD tends to reflect a situational, lower-threshold response, whereas MI represents a deeper and more enduring disruption to a person’s moral identity [4]. Burn-out, by contrast, arises from chronic occupational stress and is characterized by emotional exhaustion, depersonalization and reduced personal accomplishment [5] and is not contingent on a moral violation. MI has recently been linked to occupational exposures faced by healthcare workers (HCWs) during COVID-19, including shortages of beds, respirators and Personal Protective Equipment [6]. Williamson *et al.* (2023) found 28% of UK National Health Service (NHS) HCWs endorsed one or more items of the Moral Injury Events Scale [7], showing that exposure is common [8]. Many systemic issues highlighted by the pandemic, including lack of resources, and staff burn-out, pre-dated COVID-19 in the UK and across Europe [9]. MI can also lead to medical errors, personal and occupational dysfunction and job dissatisfaction, impacting patient care [10]. MI is also associated with increased risk of mental health problems [11], which carry their own occupational consequences. Psychiatric disorders are the most common reason for sickness absence among NHS staff in England [12]; thus, unaddressed MI may contribute to absenteeism, placing additional pressure on already stretched teams. These circumstances increase the risk of medical errors and occupational dysfunction, both affecting the quality and safety of patient care. Supporting HCWs exposed to PMIEs is therefore vital to reduce mental health problems, improve retention and prevent sickness absence.

Reflective practice (RP) is an activity during which HCWs explore their actions, feelings and decisions regarding their work often in a facilitated setting [13]. This typically involves facilitated group sessions reviewing actions and events, enabling individuals to express feelings and concerns in a supportive environment, resulting in peer support or the development of a shared, meaningful narrative. A 2021 review of 16 studies identified interventions aimed at mitigating MI in HCWs [14]: seven showed reductions in MD, others reported mixed results, although many suggested RP may provide a valuable framework for reflecting on ethical challenges.

Since COVID-19, there has been an increased focus on MI in HCWs, underscoring the need for an updated literature review. While the 2021 systematic review [14] identified various interventions for mitigating symptoms of MI among HCWs, including some with RP components, it did not focus in depth on RP as a distinct intervention. This narrative review specifically examines RP-based interventions for MI, uniquely addressing barriers to implementation, an area not covered in the earlier review.

## Methods

Ethical approval was not required as this was a literature review. Semi-structured searches were conducted in July 2024 and August 2025 across four databases, including PubMed, MEDLINE, PsycINFO and Web of Science, covering publications up to August 2025. A search strategy was co-developed between the authors using Medical Subject Headings (MeSH) terms and the Population, Intervention, Comparison and Outcome (PICO) framework [15]. We searched terms relating to ‘healthcare workers’, ‘reflective practice’ and ‘moral injury’ (see Supplementary material 1 for the full search strategy). We also searched papers including ‘military personnel’ and the ‘emergency services’ to capture evaluations of RP that may have included samples from mixed occupations. Additional articles were identified through manual citation searches.

Paper selection used inclusion criteria aligned with our aims: (i) original research with HCWs as the population and (ii) studies examining RP as an intervention for MI (including pilots, feasibility trials and randomized controlled trials [RCTs]). We defined workers in a clinical or medical setting, including administrative staff and student professionals, as HCWs. Given the emergent state of the literature, inclusion criteria were intentionally broad to encompass both clinical and non-clinical healthcare staff. Non-clinical roles were retained on the basis that patient-facing staff, such as receptionists, may nonetheless encounter morally injurious experiences, including patient aggression. We only included peer-reviewed papers in English. Exclusion criteria included (i) papers other than original research studies and (ii) papers unrelated to the investigation of RP as an intervention for MI in HCWs. Although not a systematic review, steps were taken to reduce bias in study selection. A second reviewer checked 50% of search results and screening decisions, uncertainties were discussed with the wider authorship team to promote consistency and transparency in the inclusion process. In line with narrative review methodology [16], a formal quality assessment was not conducted, but we have noted where study designs were of a higher or lower quality (e.g. pre/post-test designs versus cross-sectional reports).

## Results

In total, 12 studies met criteria (Table 1, available as Supplementary material at *Occupational Medicine* online). Most included studies sampled nurses [17–24], and four [25–28] sampled a diverse range of HCWs. Eight studies were conducted within the USA [17–19,22,23,25,26,28], the remaining in Canada [20], Denmark [27] and Norway [21,24]. The total sample size across studies was *n* = 793; individual sample sizes ranged from *n* = 13–341. Most studies (7 out of 12) had a sample size of under 50. Most interventions included structured workshops or sessions embedding RP. Please see Table 1 (available as Supplementary material at *Occupational Medicine* online) for a summary of papers related to RP interventions for reducing symptoms of MI in HCWs.

Several studies combined RP with psychoeducational or theoretical frameworks, most commonly trialled with nurses. Semler [17] trialled a 4-hour workshop with nurses, followed by two self-reflection activities. The intervention led to significant reductions in symptoms of moral distress (Moral Distress Thermometer) [29]; and improved perceived preparedness to resolve ethical conflict. However, as a quality improvement (QI) project, it lacked a control group. Similarly, Bevan and Emerson [18], piloted a three-session RP intervention over 3 weeks with 13 nurses. This was based on more philosophical psychoeducation, guided by Freirean conscientization [30], referring to developing critical insight into systemic oppression. The intervention focused on critical reflection, motivation and action, resulting in significant decrease in mean Moral Distress Scale-Revised (MDS-R) [31] score and intensity, suggesting value in embedding RP in sociopolitical and power-sensitive frameworks. Usset *et al.* [19] delivered monthly RECONN (Reflection and Connection) sessions to nurses over 6 months. There were small to moderate non-significant effects on coping, recovery, symptoms of MI and loneliness. Most participants (65–70%) found the intervention suitable. Together, these studies suggest psychoeducational RP interventions are feasible, well-received with potential to reduce symptoms of MI. However, evidence remains preliminary given reliance on small samples, self-reports and lack of control groups.

Other interventions used RP without embedding a psychoeducational component, instead focusing on more ethical dialogue. Meziane *et al.*’s [20] pilot RP intervention consisted of three 45–75-minute group sessions using structured reflection. This study used a one-group pre-test/post-test design with 19 acute care nurses and found that there was no significant difference in mean MDS-R [31] score from pre- to post-intervention. However, most nurses self-reported positive changes in their care provision and deemed the intervention feasible. The authors suggest that discrepancy between the quantitative and anecdotal findings may be due to the complex nature of the decisions acute care nurses are required to make, meaning that some HCWs may have a higher threshold for perceiving clinical effectiveness in such interventions. Given the limited sample size, however, this link would require further investigation.

Jakobsen *et al.* [21] utilized a digital ethical reflection tool to support nurses’ perceptions of the ethical challenges they encounter. The tool comprises nine questions that align with the four principles of bioethics (respect for autonomy, non-maleficence, beneficence and justice) [32]. Data were collected before and after the implementation assessing progress and effectiveness. Nurses reported an increased ethical awareness of their role. The authors also noted the tool can stimulate personal reflection and staff discourse, enhancing cohesion and strengthening managerial ethics to help decrease betrayal-related MI. These findings indicate that RP interventions without psychoeducation can enhance ethical awareness and team cohesion. The study included only qualitative data from 6 nurse leaders and 13 nurses, and findings should therefore be extrapolated cautiously.

Multisession RP interventions have also been tried with non-nurse HCWs. Kopstick *et al.* [25] tested a 90-minute workshop, including didactic presentations, role-playing scenarios, individual and group reflections involving doctors and psychologists. Self-reports indicated that the workshop is effective and feasible for enhancing trauma-informed care knowledge, which may improve care capacity and reduce symptoms of MI and burn-out. However, again, the study lacked a control group. While facilitating reflections can help mitigate symptoms of MI [33] retrospectively, a stronger evidence base for RP is needed to safely embed it as standard clinical practice.

Arquette *et al.* [26] trialled a workshop intervention with a range of HCWs (such as students, residents, fellows). This included a 75-minute interactive session, including a lecture, case-based learning, small-group and individual reflections. Thirty-four out of 60 participants completed surveys; 97% found the workshop valuable in helping them define and identify MI. This suggests that education and reflections about MI are helpful and empowering and preliminarily highlights the value of RP. However, fewer than half felt able to implement the strategies to address MI, suggesting the need to further interrogate the extent to which RP is effective. This study is similarly constrained by methodological issues, including no pre/post-test design or longitudinal measurements. More methodologically robust studies are necessary to understand the extent to which RP interventions can be effective for HCWs in reducing symptoms of MI.

Similarly, Schou-Juul *et al.* [27] evaluated the CARE intervention, which aimed to enhance HCWs’ confidence in ethical decision-making in dementia care through a reflective dialogue and shared deliberation. In one application, relatives of long-term care residents and HCWs discussed ethical issues specific to cooperation and care through two 3-hour workshops, 1 week apart. Participants engaged in ethical reflections, attentiveness to different perspectives and bioethical lectures. In the second application, HCWs completed a 2-day workshop targeting peer dialogues. Ethical issues were adapted to the context of each group and were derived from existing dementia research. Many participants reported increased confidence in navigating ethical decisions, while some reported no change due to prior expertise. While this study did not include direct MI measurement, parallels can be drawn between how RP-style workshops can be tailored to improve confidence and overall work performance and outcomes in complex, high-stake contexts.

Collectively, these studies demonstrate the adaptability of RP across healthcare groups and contexts, with preliminary signals of benefit for ethical awareness, confidence and care capacity. However, a cautious approach to interpretation is needed. Most included studies relied on small, self-selected samples, self-reports and pre/post designs without control groups, which all inflate the risk of bias and limit causal inference. Positive findings therefore reflect feasibility and acceptability rather than demonstrated efficacy. It is currently premature to draw firm conclusions about the effectiveness of RP for MI on the basis of current evidence.

We found one controlled study. Browning and Cruz [22] investigated a Reflective Debriefing session with a convenience sample of 42 intensive care unit (ICU) nurses and a non-participatory control group (*n* = 23). Both groups completed the MDS-R [31] before and after the intervention group sessions, which were facilitated by a social worker once per month over a 6-month period. Secondary quantitative analysis indicated that MI symptoms of the control group were higher than in the treatment group; however, these results were not statistically significant. Although this was the strongest study design within the review, the non-significant findings underscore the difficulty of demonstrating measurable impact, highlighting the need for adequately powered, longitudinal studies.

Given some of the promising initial results from studies exploring RP for MI, understanding the accessibility of and barriers to implementing RP is crucial to ensure that interventions are sustainable, creating long-term solutions for reducing symptoms of MI in HCWs. Even the most theoretically successful interventions will go unused if they are not accessible or practical for HCWs to participate in. See Table 2 (available as Supplementary material at *Occupational Medicine* online) for a summary of the barriers to engaging in RP.

There are personal barriers associated with engaging in RP. VanHorn *et al.*’s [23] the Minute of Silence, a guided reflective exercise for ICUs following patient deaths, had compliance rates of 2% and 17% in newly introduced units, compared to 30% where RP programmes were already established, suggesting unfamiliarity with RP as a barrier. Jakobsen *et al.*’s [21] study identified fatigue, linked to frequent organizational restructuring, and disinterest, suggesting the need to understand how RP can be better integrated in practice. A cross-sectional study of 106 health science librarians [28] found barriers, including lack of motivation, confidentiality concerns and fear of self-esteem damage or repercussions, underscoring the importance of structured training and organizational support.

Unsurprisingly, institutional barriers hindered practising RP. Jakobsen *et al.* [24] investigated how digital ethical reflection can be developed and tested to support nurses. They found that lack of manager engagement and practical applicability of information obtained through reflection may act as barriers, with participants reporting that RP was only effective or meaningful when managers paid attention to responses. Other institutional barriers include organizational culture not valuing RP [28], which can hinder its development and use.

## Discussion

This review aimed to synthesize existing studies on RP interventions for MI in HCWs. The evidence identified appears to be broadly supportive of the use of RP within healthcare, though the evidence base remains in its infancy.

Although previous reviews have focused on interventions for MI in HCWs [14], or MI in clinical practice [10,34,35], we believe this is the first review to explore RP as a targeted response to MI in healthcare, making it a timely and necessary contribution to the field. The choice of narrative review can be a strength because it is useful for summarizing literature on topics such as this where the evidence is sparse [36]. While we did not conduct formal quality assessments of each included study, our methods were otherwise similar to a systematic review [37].

Many of the papers in this review, while helpful in improving understanding of MI and evaluating what works, for whom and why, are limited due to not including a control group or being small-scale pilot studies of RP as a MI intervention—only one study [22] included a control group. Small convenience samples limit representativeness, so findings should be interpreted cautiously when generalizing widely. RCTs with longitudinal design, including a control group, are necessary to ascertain the sustained impact of RP as a means of resolving symptoms of MI. Future research should include other essential non-clinical team members such as administrative staff, cleaners and volunteers, who were typically not included in the studies mentioned. Due to the limited research in this field, we included administrative staff (such as health science librarians) in our review, to capture a wide range of perspectives. While there is evidence that HCWs in patient-facing roles [38] were more likely to report probable mental disorders during COVID-19, there is little research on MI. Patient-facing roles can also include non-clinical roles such as receptionists, so for this review, we had broad inclusion criteria. Further, it is possible that healthcare-adjacent staff may also experience PMIEs such as feelings of organizational betrayal. Future research should investigate which groups may be more at risk of MI and the mechanisms for this, how to successfully embed RP and address barriers for implementation and engagement. In addition, although MI has been linked to patient care [10], very few studies in our review investigate this relationship directly—future studies could focus on how MI impacts patient care more explicitly.

We found 12 studies in total, eight sampling nurses and the rest other HCWs. All studies were conducted in the USA, Canada, Denmark or Norway. The results show preliminary support for RP as an intervention for MI in HCWs. Two studies [17,18] showed significant reductions in MD scores; one [20] found no statistically significant changes, although most nurses said they would recommend the intervention. Another [22] found the pre/post-test was limited by small sample sizes and did not show significant differences between scores in the experimental and control groups, despite participants unanimously reporting benefits in the feedback survey. Similarly, using a pre/post-test design, while not statistically significant, small to moderate effects were found in RECONN for improving symptoms of MI [19]. Self-reports from remaining studies [21,23–28], although lacking pre/post-test design, suggested HCWs benefited from RP interventions for MI/confidence in ethical decisions. Existing evidence, while preliminary and lacking methodological robustness, appears to support integrating RP as a way of mitigating MI for HCWs.

The findings have implications for establishing RP in healthcare; we found that even personal barriers such as lack of familiarity and time are a product of systemic pressures in busy clinical environments. Thus, the onus of repairing symptoms of MI should be with organizations rather than individuals. The presence of such barriers may potentially be mitigated if there were protected time and multiple opportunities for engaging in RP [23]. For example, familiarity with RP can be gained through repeated exposure to RP [23]. Addressing cultural resistance, like the perception of RP as an illegitimate tool, may demand a shift in organizational priorities too. Specifically, creating safe, non-judgmental spaces for reflection may help alleviate personal fears of vulnerability or repercussions, empowering HCWs to engage meaningfully with RP.

## Supplementary Material

kqag060_Supplementary_Data

## Open access

For the purposes of open access, the author has applied a Creative Commons Attribution (CC BY) licence to any Accepted Author Manuscript version arising from this submission.
