# Supplementary material for: Reflective practice to reduce moral injury in healthcare workers
Source: Occup Med (Lond). 2026 Sep 17;76(6):435–41. doi: 10.1093/occmed/kqag060 (PMC13673504; doi:10.1093/occmed/kqag060)
Supplement: Supplementary file 1 [file kqag060_supplementary_data.zip › 25-OP-159.R3a FINAL supplementary tables.doc]

Table 1. Characteristics of studies included in the review

| **Reference** | **Country** | **Aim** | **Sample size** | **Intervention components** | **Summary of results for MI** |
| --- | --- | --- | --- | --- | --- |
| Semler (2022) (1) | United States | To decrease critical care nurses’ experiences of MI following the intervention. | 18 nurses | Four-hour workshop (using the Knowledge to Action/KTA; (2) framework, followed by two individual self-reflection activities at 2-3 weeks and 5-6 weeks after the workshop in order to monitor use of framework. | Significant decrease in moral distress, measured by the Moral Distress Thermometer (MDT; (3) following the workshop intervention  Increased ability to identify conflicting values, knowledge of role expectations, and perceived preparedness to resolve ethical conflict. |
| Bevan and Emerson (2020) (4) | United States | To evaluate feasibility and acceptability of a “conscientization” intervention for critical care nurses who have recently experienced MI. | 13 nurses | Composed of three consecutive group sessions:  1) Critical Reflection: recognising and talking about social inequalities and MI  2) Critical Motivation: guiding the participants collectively to identify their agency  3) Critical Action: learning from other participants about taking action | Significant decrease in the mean moral distress score (measured by the Moral Distress Scale-Revised/MDS-R) following the intervention (5).  Significant decreases in moral distress intensity following the intervention |
| Usset et al., (2024) (6) | United States | To assess feasibility, acceptability and preliminary effectiveness of RECONN (Reflection and Connection). | 17 Medical Intensive Care Unit (MICU) Nurses. | Six months of RECONN sessions, held once a month. RECONN was designed using an Evidence-Based Quality Improvement (EBQI) process. The sessions focused on identifying personal events and the wider group reflecting and providing feedback (e.g., validation, deeper understanding, guidance). | Between 65-70% of nurses found RECONN to be fitting, applicable and doable for nurses. Small to moderate effects for RECONN improving coping, MI, loneliness and emotional recovery were found. |
| Meziane et al., (2018) (7) | Canada | To assess feasibility, acceptability, and preliminary effects of an RP intervention on MI. | 19 acute care nurses | Three 45–75-minute group sessions using a structured reflection model. | 16/19 nurses completed the intervention  13/16 noticed self-reported changes in their practice following the intervention  No significant difference in the mean of the pre- and post-intervention moral distress as measured by the MDS-R (5). |
| Jakobsen et al., (2024) (8) | Norway | To explore the experiences of nurses and nurse leaders in using digital ethical reflection as a tool for ethics work in home nursing care. | 19 nurses and six nurse leaders | A digital registration form which nurses can use during or after a shift. It contains 9 short questions that aim to facilitate ethical reflection. | Four key themes: a constant walk on the edge between engagement and discouragement, lost in translation, tuning in to the ethical dimension & navigating ethical uncertainties.  Nurses reported greater awareness and normalisation of the ethics involved in day-to-day tasks, which can contribute to a level of reduction in moral distress. |
| Browning and Cruz (2018) (9) | United States | To develop and test a protocol for alleviating MI through regular, social worker-facilitated debriefings with ICU nursing staff. | 42 ICU nurses:  23 in the control group and  19 in the experimental group. | Social worker-facilitated Reflective Debriefing protocol, including an educational component and guided reflection. | Pre/post-tests were limited by a small sample size and as a result, were non-significant. Participants from the experimental group unanimously reported benefits from the debriefings, with 100% requesting to continue. |
| VanHorn et al., (2020) (10) | United States | To improve ICU nurses’ emotional coping skills through the use of a nurse-guided reflective exercise immediately following a patient's death and to explore the impact of RP on the nurses’ emotional and moral distress. | 341 nurses employed across three adult ICUs | ‘A Minute’s Silence’ comprises dialogue and recitation to guide nurses’ words during the reflective session and additional notes for guidance. It is structured to be completed within one minute to balance the need for reflection with nurses’ clinical workload. | Compliance with RP after patient death was low (17% in MS-ICU). Nurses reported neutral to positive emotions after the exercise, a few felt drained, suggesting promise in reducing distress and supporting emotional processing. Sustainable education, support and attention to barriers are required for the RP to be adopted successfully. |
| Jakobsen et al., (2023) (11) | Norway | To investigate how digital ethical reflection can support ethics work among nurses working in long-term care. | 17 nurses, 14 of which took part in the focus group | Digital ethical reflection tool aimed to enable employees to effectively identify and report ethical challenges and provide managers insight into the ‘ethical temperature’ of the department. The tool comprised of 9 questions. | 64% of nurses wanted the department to devote time to reflection on perceived ethical issues. Many had increased attention to morally charged situations and also reported that engagement in the tool was only meaningful if the manager pays attention to responses. |
| Kopstick et al., (2025) (12) | United States | To create an “introductory primer” for increasing HCWs’ capacity to increase trauma informed care (TIC) as a universal precaution. | 80 HCWs in various roles (physicians, nurses, research coordinators, and hospital administrators); 24 participants completed the pre-post knowledge surveys. | 90-minute workshop on TIC featuring didactics, role-playing, writing and  self-reflection exercises, and large-group debriefings. | Participants experienced a significant increase in self-perceived TIC knowledge, which in turn can increase their capacity to care, thus decreasing MI and alleviating burnout. |
| Arquette et al., (2023) (13) | United States | Aim of the workshop was to define MI, identify it, and develops tools to combat or mitigate MI. | 60 HCWs (residents, fellows, attending physicians, and program administrators from residency programs within Stanford and other US and international institutions) attended; 34 participants responded to the questionnaire. | 75-minute workshop which involved didactic session focused on defining MI, identifying one's core values, identifying personal examples of MI and sharing them in a small-group setting, identifying tools/strategies to help combat MI. | 97% of these participants reported they found the workshop valuable as it helped them define and identify MI.  100% would recommend the workshop to a friend or colleague.  Almost half felt they could implement strategies to address MI after participating in the workshop. |
| Schou-Juul et al., (2025) (14) | Denmark | To evaluate the impact of the CARE intervention on healthcare professionals’ perceived confidence in ethical decision-making in dementia care | 55 participants, 2 groups. Group 1: relatives of residents in long-term care & healthcare professionals n=25 Group 2: Healthcare professionals only n=30 Backgrounds were diverse but skewed towards social and health care helpers/assistants (n=31) | CARE intervention included a structured agenda led by a moderator experienced in dementia care.  Group 1: Two 3-hour workshops one-week apart.  Intervention focused on ethical issues, decision-making, ethical reflection and bioethical lectures.  Group 2: Two-day workshop. Intervention focused on an ethics course and peer-dialogues. | Five themes across two overarching domains: the perceived influence of the CARE intervention on healthcare professionals’ confidence and factors impacting their confidence.  The intervention reinforced confidence for many, especially through peer validation, shared reflection and interaction with families.  Some participants reported no change due to pre-existing high confidence levels. |
| Miller (2020) (15) | United States | To examine the use of RP among health sciences librarians, perceived benefits and perceived barriers to use. | 106 librarians | Self-report via 2014 Greenall and Sen questionnaire adapted for health science librarians.  Questions included RP engagement, differences in training vs practice, benefits and barriers. | 87% of participants reported they consciously spent time reflecting. Respondents identified a wide variety of benefits of RP, as well as barriers, which included lack of time, knowledge or experience. |

**References**

1.	Semler LR. Moral distress to moral success: Strategies to decrease moral distress. Nurs Ethics. 2023 Feb 1;30(1):58–70.

2.	Graham ID, Logan J, Harrison MB, Straus SE, Tetroe J, Caswell W, et al. Lost in knowledge translation: Time for a map? Journal of Continuing Education in the Health Professions. 2006;26(1):13–24.

3.	Wocial L, Weaver MT. Development and psychometric testing of a new tool for detecting moral distress: the Moral Distress Thermometer. Journal of Advanced Nursing [Internet]. 2012 [cited 2025 Dec 22]; Available from: https://onlinelibrary.wiley.com/doi/10.1111/j.1365-2648.2012.06036.x

4.	Bevan NA, Emerson AM. Freirean Conscientization With Critical Care Nurses to Reduce Moral Distress and Increase Perceived Empowerment: A Pilot Study. Advances in Nursing Science. 2020 Sept;43(3):E131.

5.	Hamric AB, Borchers CT, Epstein EG. Development and Testing of an Instrument to Measure Moral Distress in Healthcare Professionals. AJOB Primary Research. 2012 Apr;3(2):1–9.

6.	Usset TJ, Godzik C, Harris JI, Wurtz RM, Pyne JM, Edmonds SW, et al. Building Social Support and Moral Healing on Nursing Units: Design and Implementation of a Culture Change Intervention. Behavioral Sciences. 2024 Sept;14(9):796.

7.	Meziane D, Ramirez-Garcia MP, Fortin ML. A reflective practice intervention to act on the moral distress of nurses providing end-of-life care on acute care units. Int J Palliat Nurs. 2018 Sept 2;24(9):444–51.

8.	Jakobsen L, Olsen RM, Brinchmann BS, Devik SA. Digital ethical reflection in home nursing care: Nurse leaders’ and nurses’ experiences [Internet]. 2024 [cited 2025 Dec 22]. Available from: https://journals.sagepub.com/doi/10.1177/09697330241244543

9.	Browning ED, Cruz JS. Reflective Debriefing: A Social Work Intervention Addressing Moral Distress among ICU Nurses. Journal of Social Work in End-of-Life & Palliative Care. 2018 Jan 2;14(1):44–72.

10.	VanHorn EK, Worobel-Luk P, Tanner B, Jang H. Nurse-facilitated reflective exercise post patient death in the ICU: a best practice implementation project. JBI Evidence Implementation. 2021 June;19(2):162.

11.	Jakobsen L, Olsen RM, Brinchmann BS, Devik SA. Developing and Testing Digital Ethical Reflection in Long-Term Care: Nurses’ Experiences. Sage Open Nursing. 2023 Jan 1;9:23779608221150725.

12.	Kopstick AJ, Aly AM, Zientek E, Williams CN, Hall TA, Macauley RC, et al. Trauma-Informed Care as a Universal Precaution: A Brief, Case-Based, Educational Primer Featuring Role-Playing and Individual Self-Reflection Exercises. J Contin Educ Health Prof. 2025 Jan 1;45(1):63–6.

13.	Arquette C, Peicher V, Ajayi A, Alvarez D, Mao A, Nguyen T, et al. Moral Injury: How It Affects Us and Tools to Combat It. MedEdPORTAL. 2023;19:11357.

14.	Schou-Juul F, Tjørnhøj-Thomsen T, Lauridsen S. Enhancing healthcare professionals’ confidence in ethical decision-making when caring for people with dementia: A qualitative evaluation of the CARE intervention. Dementia. 2025 Nov 1;24(8):1581–99.

15.	Miller JM. Reflective practice and health sciences librarians: engagement, benefits, and barriers. Journal of the Medical Library Association. 2020 Jan 2;108(1):17–28.

Table 2. Summary of barriers to engaging in RP identified in the included studies.

| **Personal barriers** | **Institutional barriers** |
| --- | --- |
| - Lack of familiarity with RP (1) - Fatigue (2) - Disinterest (3) - Lack of experience (3) - Lack of motivation (4) - Concerns about confidentiality (4) - Absence of perceived benefits (4) - Fear of damaging one's self-esteem (4) - Unwillingness to focus on emotions or admit mistakes (4) - Fear of potential repercussions (4) | - Lack of managerial support (3) - Lack of practical applicability (3) - Time pressure in busy clinic clinical environments (1,3,4) - Organisational hierarchy portraying RP as an abnormal practice (3) - Organisational culture not valuing RP (4) |

**References**

1.	VanHorn EK, Worobel-Luk P, Tanner B, Jang H. Nurse-facilitated reflective exercise post patient death in the ICU: a best practice implementation project. JBI Evid Implement. 2021 June;19(2):162.

2.	Jakobsen L, Olsen RM, Brinchmann BS, Devik SA. Digital ethical reflection in home nursing care: Nurse leaders’ and nurses’ experiences [Internet]. 2024 [cited 2025 Dec 22]. Available from: https://journals.sagepub.com/doi/10.1177/09697330241244543

3.	Jakobsen L, Olsen RM, Brinchmann BS, Devik SA. Developing and Testing Digital Ethical Reflection in Long-Term Care: Nurses’ Experiences. Sage Open Nurs. 2023 Jan 1;9:23779608221150725.

4.	Miller JM. Reflective practice and health sciences librarians: engagement, benefits, and barriers. J Med Libr Assoc. 2020 Jan 2;108(1):17–28.
