# Supplementary material for: Reflective practice to reduce moral injury in healthcare workers
Source: Occup Med (Lond). 2026 Sep 17;76(6):435–41. doi: 10.1093/occmed/kqag060 (PMC13673504; doi:10.1093/occmed/kqag060)
Supplement: Supplementary file 1 [file kqag060_supplementary_data.zip › 25-OP-159.R3 Supplementary_Material_Revised.doc]

# Supplementary material 1

| **Key word** | **Terms** |
| --- | --- |
| Reflective practice | **“Reflection”**  **OR**  **“Reflective practice*”**  **OR**  **“Reflection Process”**  **OR**  **“Reflection Processes”** |
| Moral injury | **“Moral Injury”**  **OR**  **“Moral distress”**  **OR**  **“Ethical stress”**  **OR**  **“Ethical injury”**  **OR**  **“Moral dissonance”**  **OR**  **“Spiritual injury”**  **OR**  **“Conscience violation”**  **OR**  **“Moral disillusionment”** |
| Healthcare | **“Healthcare”**  **OR**  **“healthcare worker*”**  **OR**  **“Doctor*”**  **OR**  **“Nurse*”**  **OR**  **“NHS”**  **OR**  **“National health service”**  **OR**  **“Health Care Provider*”**  **OR**  **“Health Care Provider*”**  **OR**  **“Health Care Professional*”** |
| Emergency services | **“Emergency service*”**  **OR**  **“Police”**  **OR**  **“Ambulance*”**  **OR**  **“Firefighters*”**  **OR**  **“Paramedic*”** |
| Military | **“Military service*”**  **OR**  **“Soldiers”**  **OR**  **“Armed forces”**  **OR**  **“Veteran*”**  **OR**  **“service person*”**  **OR**  **“military personnel”**  **OR**  **Army**  **OR**  **Royal Air Force**  **OR**  **RAF**  **OR**  **Royal Navy**  **OR**  **Navy**  **OR**  **Air force**  **OR**  **Forces** |

## Specific database search strategy

| **Database** | **Terms used** | **Filters Used** |
| --- | --- | --- |
| PubMed | All above terms for reflective practice, moral injury AND (one of the 3 working population)  Reflection Block:  ("reflection"[Title/Abstract] OR "reflective practice*"[Title/Abstract] OR  "reflection process"[Title/Abstract] OR "reflection processes"[Title/Abstract])  AND  Moral Injury Block:  ("moral injury"[Title/Abstract] OR "moral distress"[Title/Abstract] OR  "ethical stress"[Title/Abstract] OR "ethical injury"[Title/Abstract] OR  "moral dissonance"[Title/Abstract] OR "spiritual injury"[Title/Abstract])  Population Block:  Healthcare:  ("healthcare"[Title/Abstract] OR "healthcare worker*"[Title/Abstract] OR  doctor*[Title/Abstract] OR nurse*[Title/Abstract] OR NHS[Title/Abstract] OR  "national health service"[Title/Abstract] OR "health care provider*"[Title/Abstract]  OR "health care professional*"[Title/Abstract])  Emergency Services:  ("emergency service*"[Title/Abstract] OR police[Title/Abstract] OR  ambulance*[Title/Abstract] OR firefighter*[Title/Abstract] OR  paramedic*[Title/Abstract])  Military:  ("military service*"[Title/Abstract] OR soldiers[Title/Abstract] OR  "armed forces"[Title/Abstract] OR veteran*[Title/Abstract] OR  "service person*"[Title/Abstract] OR "military personnel"[Title/Abstract] OR  army[Title/Abstract] OR "royal air force"[Title/Abstract] OR RAF[Title/Abstract] OR  "royal navy"[Title/Abstract] OR navy[Title/Abstract] OR  "air force"[Title/Abstract] OR forces[Title/Abstract])  #1 AND #2 AND (#3 OR #4 OR #5) | English Language |
| Medline | (reflect*.ti,ab.  OR reflective practice.ti,ab.  OR reflection process*.ti,ab.)  AND  (moral injury.ti,ab.  OR moral distress.ti,ab.  OR moral dissonance.ti,ab.  OR ethical stress.ti,ab.  OR ethical injury.ti,ab.  OR spiritual injury.ti,ab.  OR Moral Injury/)  AND  (healthcare worker*.ti,ab.  OR health care professional*.ti,ab.  OR healthcare.ti,ab.  OR doctor*.ti,ab.  OR nurse*.ti,ab.  OR NHS.ti,ab.  OR national health service.ti,ab.  OR Health Personnel/ )  OR  (emergency service*.ti,ab.  OR police.ti,ab.  OR paramedic*.ti,ab.  OR firefighter*.ti,ab.  OR ambulance*.ti,ab.  OR Emergency Responders/)  OR  ( military.ti,ab.  OR armed forces.ti,ab.  OR veteran*.ti,ab.  OR soldiers.ti,ab.  OR service person*.ti,ab.  OR military personnel.ti,ab.  OR army.ti,ab.  OR navy.ti,ab.  OR air force.ti,ab.  OR RAF.ti,ab.  OR Veterans/  OR Military Personnel/) | English Language |
| PsychINFO (Ovid) | (reflect*.ti,ab.  OR reflective practice.ti,ab.  OR reflection process*.ti,ab.)  AND  (moral injury.ti,ab.  OR moral distress.ti,ab.  OR moral dissonance.ti,ab.  OR ethical stress.ti,ab.  OR ethical injury.ti,ab.  OR spiritual injury.ti,ab.  OR Moral Injury/)  AND  (healthcare worker*.ti,ab.  OR health care professional*.ti,ab.  OR healthcare.ti,ab.  OR doctor*.ti,ab.  OR nurse*.ti,ab.  OR NHS.ti,ab.  OR national health service.ti,ab.  OR Health Personnel/ )  OR  (emergency service*.ti,ab.  OR police.ti,ab.  OR paramedic*.ti,ab.  OR firefighter*.ti,ab.  OR ambulance*.ti,ab.  OR Emergency Responders/)  OR  ( military.ti,ab.  OR armed forces.ti,ab.  OR veteran*.ti,ab.  OR soldiers.ti,ab.  OR service person*.ti,ab.  OR military personnel.ti,ab.  OR army.ti,ab.  OR navy.ti,ab.  OR air force.ti,ab.  OR RAF.ti,ab.  OR Veterans/  OR Military Personnel/) | English Language |
| Web of Science | Topic Search= (reflect*  OR "reflective practice"  OR "reflection process*")  AND  Topic Search =("moral injury"  OR "moral distress"  OR "moral dissonance"  OR "ethical stress"  OR "ethical injury"  OR "spiritual injury")  AND  Topic Search =( "healthcare worker*"  OR "health care professional*"  OR healthcare  OR doctor*  OR nurse*  OR NHS  OR "national health service")  OR  ("emergency service*"  OR police  OR paramedic*  OR firefighter*  OR ambulance* )  OR  ( military  OR "armed forces"  OR veteran*  OR soldiers  OR "service person*"  OR "military personnel"  OR army  OR navy  OR "air force"  OR RAF) | English Language |
